# Twisted Carotenoids Do Not Support Efficient Intramolecular Singlet Fission in the Orange Carotenoid Protein

**DOI:** 10.1021/acs.jpclett.3c01139

**Published:** 2023-06-26

**Authors:** George A. Sutherland, James P. Pidgeon, Harrison Ka Hin Lee, Matthew S. Proctor, Andrew Hitchcock, Shuangqing Wang, Dimitri Chekulaev, Wing Chung Tsoi, Matthew P. Johnson, C. Neil Hunter, Jenny Clark

**Affiliations:** † Plants, Photosynthesis and Soil, School of Biosciences, University of Sheffield, Sheffield S10 2TN, U.K.; ‡ Department of Physics and Astronomy, University of Sheffield, Sheffield S3 7RH, U.K.; § SPECIFIC, Faculty of Science and Engineering, Swansea University, Swansea SA1 8EN, U.K.; ∥ Department of Chemistry, University of Sheffield, Sheffield S3 7HF, U.K.

## Abstract

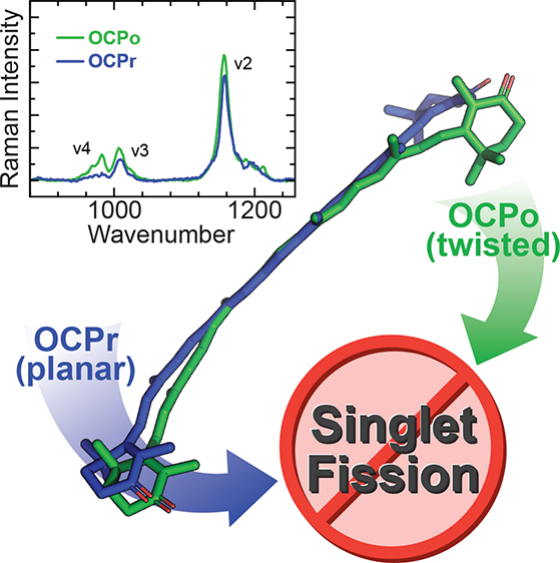

Singlet exciton fission is the spin-allowed generation of two triplet electronic excited states from a singlet state. Intramolecular singlet fission has been suggested to occur on individual carotenoid molecules within protein complexes provided that the conjugated backbone is twisted out of plane. However, this hypothesis has been forwarded only in protein complexes containing multiple carotenoids and bacteriochlorophylls in close contact. To test the hypothesis on twisted carotenoids in a “minimal” one-carotenoid system, we study the orange carotenoid protein (OCP). OCP exists in two forms: in its orange form (OCPo), the single bound carotenoid is twisted, whereas in its red form (OCPr), the carotenoid is planar. To enable room-temperature spectroscopy on canthaxanthin-binding OCPo and OCPr without laser-induced photoconversion, we trap them in a trehalose glass. Using transient absorption spectroscopy, we show that there is no evidence of long-lived triplet generation through intramolecular singlet fission despite the canthaxanthin twist in OCPo.

Singlet exciton fission (SF) is the conversion of a spin-0 singlet exciton^1^ (or excited singlet state) into a pair of spin-1 triplet excitons.^2−5^ This multiexciton generation process has been studied over the past decade primarily because of its promise to improve solar cell efficiency;^6−10^ one high-energy photon creates two low-energy excited states, which could be harvested by conventional photovoltaic devices in a process minimizing energetic losses due to thermalization. SF has other potential applications for nonlinear optics,^11−13^ organic light-emitting diodes,^14^ or even quantum technologies^15−18^ by taking advantage of the virtue that a single photon creates a pair of spin-entangled quantum states. However, despite promising results,^19,20^ practical applications have yet to be realized, in part due to the limited library of materials that undergo SF, none of which is yet ideal.^4,7^

In the search for other SF materials, the polyenes, “class III” SF materials according to Smith and Michl’s categorization,^2^ form an intriguing materials class. In these materials, the lowest-lying singlet excited state (S_1_) has dominant triplet-pair character, denoted ^1^(TT) (see refs (3), (21), and (22)), and thus demonstrates negligible one-photon absorption from the ground state. S_1_ is instead accessed by internal conversion following excitation to the strongly absorbing S_2_ state.

This SF class includes conjugated polymers such as polydiacetylene,^23−25^ poly(alkylthienylenevinylene),^26−28^ a new generation of donor–acceptor singlet fission polymers,^29−32^ quinoidal thiophenes,^33−37^ carbene-based diradicaloids,^38^ and antiaromatic core-structured molecules.^39,40^ The polyene family also includes the carotenoids, a large class of over 1000 naturally occurring molecules,^41,42^ represented here by canthaxanthin (CAN), which forms the subject of this work (see structure in Figure 1a).

**Figure 1:**
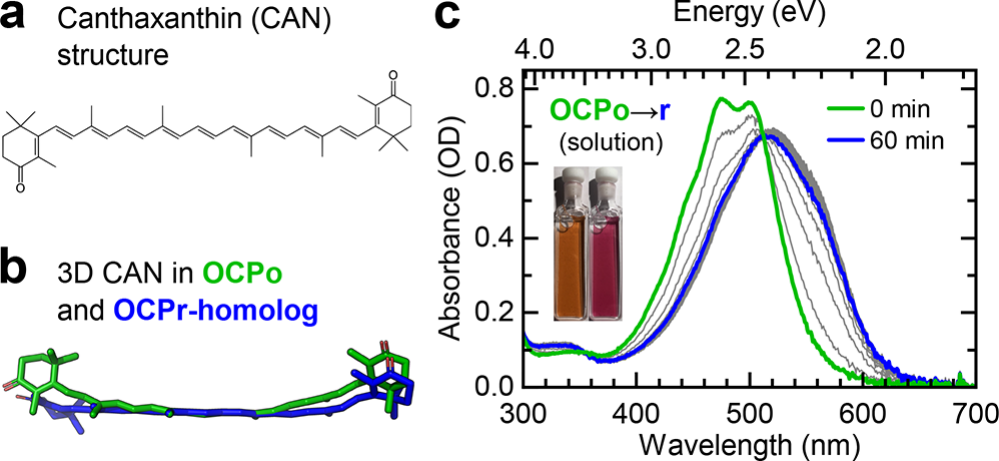
**The orange carotenoid protein (OCP) photoswitches from orange (OCPo) to red (OCPr) forms with different carotenoid conformations.** The OCP studied here binds a single CAN carotenoid whose skeletal structure is shown in (a). The bound CAN conformation depends on the OCP form, as shown in (b): when CAN is bound in OCPo (green, data from X-ray diffraction structure, PDB entry 4XB5(43,44)), it has a twisted conformation; when bound in an OCPr N-terminal homologue (blue, data from X-ray diffraction structure of red carotenoid protein (RCP), PDB entry 4XB4(43,45)), it is planar. (c) In solution, OCPo converts to OCPr under white-light illumination (1600 μmol of photons m^–2^ s^–1^), resulting in a change in color (see the inset) and absorbance spectrum (main panel). The spectra were taken in 1 min intervals under constant white-light illumination. In the dark, OCPr converts back to OCPo (see Figure S1). The optical path length for solution measurements was 1 mm.

In comparison with better-studied “class I” SF materials,^2,46−49^ mostly based on molecules such as pentacene^50,51^ or tetracene,^52−55^ SF in polyenes is less well understood. This is attributable in part to their complex manifold of low-lying triplet-pair states^56−58^ and strong vibronic coupling^59,60^ and also partly due to the sensitivity of the photophysics to conjugation length and molecular geometry. In polyenes, the lowest-lying ^1^(TT) state that makes up the dominant contribution of S_1_^21,22,61,62^ contains tightly bound triplets that are unlikely to easily separate into free triplets^63^ without additional energy.^56^

Indeed, while intramolecular singlet fission (iSF) has been observed in a variety of long-chain polyenes in solution,^23−32^ unlike the recently designed “class I” iSF systems,^47−49,64^ the triplet pairs in polyenes decay rapidly (ps–ns) to the S_0_ ground state.^3^ Even in carotenoid aggregates, where intermolecular SF occurs between neighboring chromophores,^65−70^ the majority of triplet excited states decay to S_0_ surprisingly quickly (within a nanosecond).^3,65,70^ In isolated carotenoids in solution, the dominant deactivation channel from the photoexcited S_2_ state is internal conversion to S_1_. To our knowledge, there is no evidence that isolated carotenoids in solution demonstrate iSF.

Nevertheless, similarly to recent reports that torsion or twisting along a molecular backbone can allow both rapid iSF and formation of long-lived triplets in “class I” SF materials,^48,64^ iSF along a single *twisted* carotenoid chain to produce long-lived (μs) triplets has been suggested to occur in some photosynthetic light-harvesting complexes (LHCs).^71−75^ In these systems, the protein binds the carotenoid, so that it is constrained in a twisted geometry. This twist reportedly stabilizes a triplet at either end of the molecule.^71,73^

This hypothesis was initially proposed to explain the presence of SF in the light-harvesting antenna (LH1) from *Rhodospirillum rubrum* because of the large intermolecular distances between neighboring carotenoids (>10 Å).^71^ More recently, Yu et al.^73^ observed a correlation between the presence of SF and the so-called ν_4_ resonance Raman peak (∼980 cm^–1^) in LHCs (LH1-RC and LH2) from *Thermochromatium tepidum* and *Rhodobacter sphaeroides* 2.4.1. The intensity of ν_4_ is related to carotenoid backbone twisting,^73,76^ so this finding led to the conclusion that backbone twisting of the carotenoid is the “structural determinant” that enables iSF.^73^

To test the hypothesis that SF can occur along a single twisted carotenoid chain, we examined a protein that binds a single carotenoid: the orange carotenoid protein (OCP). In OCP the protein exists in two forms, orange (OCPo) and red (OCPr), with the carotenoid in either a twisted or planar conformation, respectively (see Figure 1). By studying both forms with the protein fixed in a trehalose–sucrose glass, we demonstrate that a twisted backbone is not sufficient to enable iSF in a protein-bound carotenoid. In light of recent work understanding magnetic field effects (MFEs) in SF systems,^77,78^ we also discuss published reports of MFEs in LHCs from purple bacteria^79−84^ and find that the reported MFEs are also inconsistent with iSF. Overall, we conclude that iSF is not supported on carotenoids bound to the OCP and is unlikely to occur in LHCs.

In this study, the OCP was produced in *Escherichia coli* by virtue of a dual plasmid system comprising pET28a with the *Synechocystis* sp. PCC 6803 OCP gene (slr1963) and pAC-CANTHipi, which provides near-100% accumulation of CAN.^85^ Carotenoid-containing protein was isolated according to the method described in Supporting Information (SI) section S1.1.

In solution, upon illumination with white light, the dark-adapted OCPo form undergoes a conformational switch to the OCPr form, with a concomitant red shift of its steady-state absorbance spectrum due to the effective conjugation length extension of the bound carotenoid^76,86−88^ (see Figure 1c). The change is reversible, with back-conversion from OCPr to OCPo occurring in the dark (see Figure S1).

Previously published X-ray diffraction structures by Leverenz, Sutter, and co-workers^43^ show that the conjugated backbone of the bound carotenoid is twisted out of the plane of conjugation in OCPo (PDB entry 4XB5(44)), while in OCPr N-terminal domain homologues such as red carotenoid protein (RCP) it is relatively planar (PDB entry 4XB4(45)). The difference between the two conformations of CAN is depicted in Figure 1b using data from X-ray diffraction structures.^43^ The different protein conformations containing a twisted and nontwisted form of CAN provide an uncomplicated model system to study the role of carotenoid geometry on iSF.

To avoid the problems associated with using spectroscopy to probe a light-activated conformational switch, we prevent the conformational change by trapping the protein in either its OCPo or OCPr conformation in a trehalose–sucrose glass as previously described.^70^ This glass matrix prevents OCPo ⇄ OCPr conversion, as demonstrated in Figure 2a,b, and allows us to probe each conformation in isolation at room temperature without altering its conformation or photophysics.^89^

**Figure 2:**
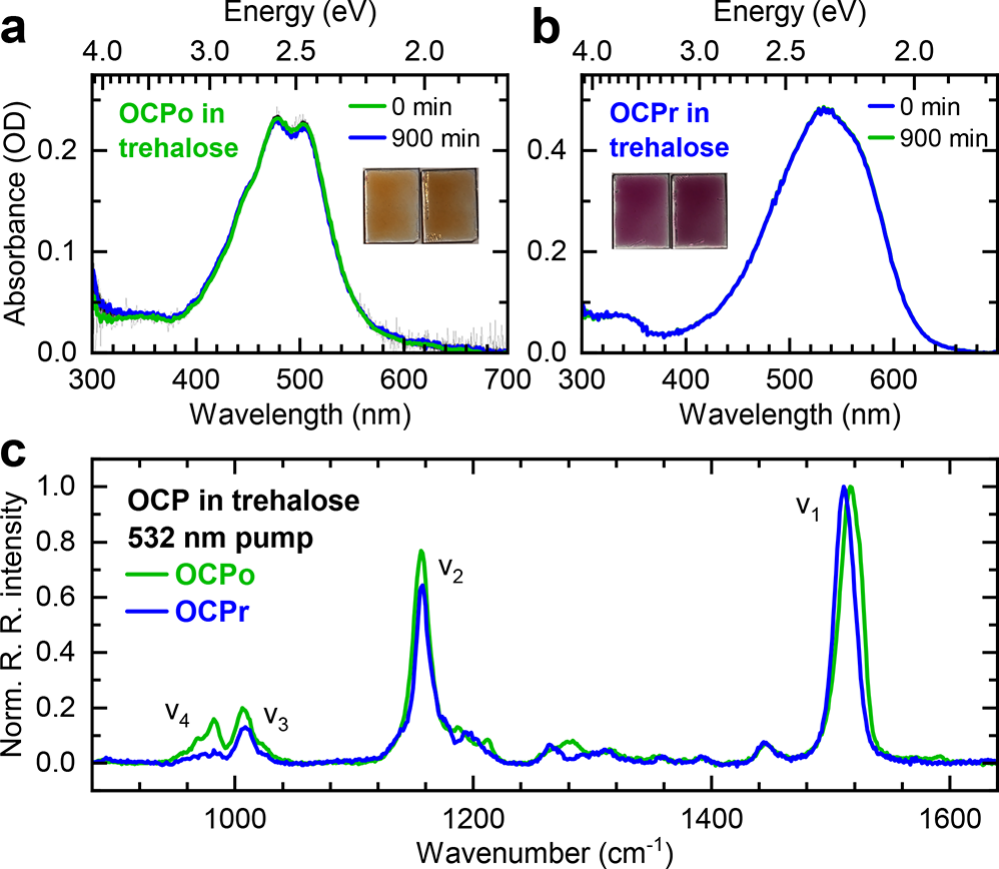
**OCPo and OCPr trapped in trehalose glass films.** The steady-state absorbance spectra of the OCPo film (a) were taken in 1 min intervals under constant white-light illumination (1600 μmol of photons m^–2^ s^–2^), and the spectra of the OCPr film (b) were taken in 1 min intervals at 22 °C in darkness. No changes in spectra were observed over 900 min. (c) The resonance Raman spectra of OCPo (green) and OCPr (blue) films in trehalose glass show vibrational peaks typical of carotenoids, labeled following convention. The spectra show a significant difference in the intensity of the ν_4_ vibrational peak between OCPo and OCPr and a shift of the ν_1_ peak. The Raman measurements were performed by using a 532 nm laser. Data are averages of two successive scans and normalized to the peak ν_1_ intensity.

To confirm the twisted/planar conformations of CAN in the OCPo/OCPr glass films, we turn to resonance Raman spectroscopy. As described above,^73,76^ the presence of a so-called ν_4_ peak at ∼980 cm^–1^ in the resonance Raman spectrum of carotenoids (due to out-of-plane C–H wagging modes^90^) is generally associated with a backbone twist of the carotenoid.^73,76^Figure 2c shows the resonance Raman spectra of OCPo (blue) and OCPr (green). Consistent with previous measurements on an echinenone-binding OCP,^76^ we observe a larger twist-induced ν_4_ peak in OCPo than in OCPr, confirming that the native geometry is maintained in trehalose-encapsulated OCPo and OCPr.

Having established that the CAN backbone is more twisted in OCPo than in OCPr, we test the suggestion that such a twist is the determinant for iSF reactivity.^72,73,75^ Picosecond transient absorption spectra and dynamics are shown in Figures 3 and 4, respectively. Global lifetime analysis of the data is shown in Figures S5 and S6, but simply from inspection of the raw data in Figure 3 we see that all spectral features in both OCPo (green) and OCPr (blue) decay to <1% of the initial population within 30 ps. Importantly, we observe no obvious formation of SF-generated triplets. Instead, both OCPo and OCPr broadly demonstrate the expected isolated carotenoid behavior characterized by rapid internal conversion from S_2_ to S_1_ (as evidenced by the instrument-limited decay of an excited-state absorption (ESA) in the near-infrared region) and subsequent decay of S_1_-like states to the ground state. Therefore, a twist along the carotenoid backbone is not sufficient to enable iSF.

**Figure 3:**
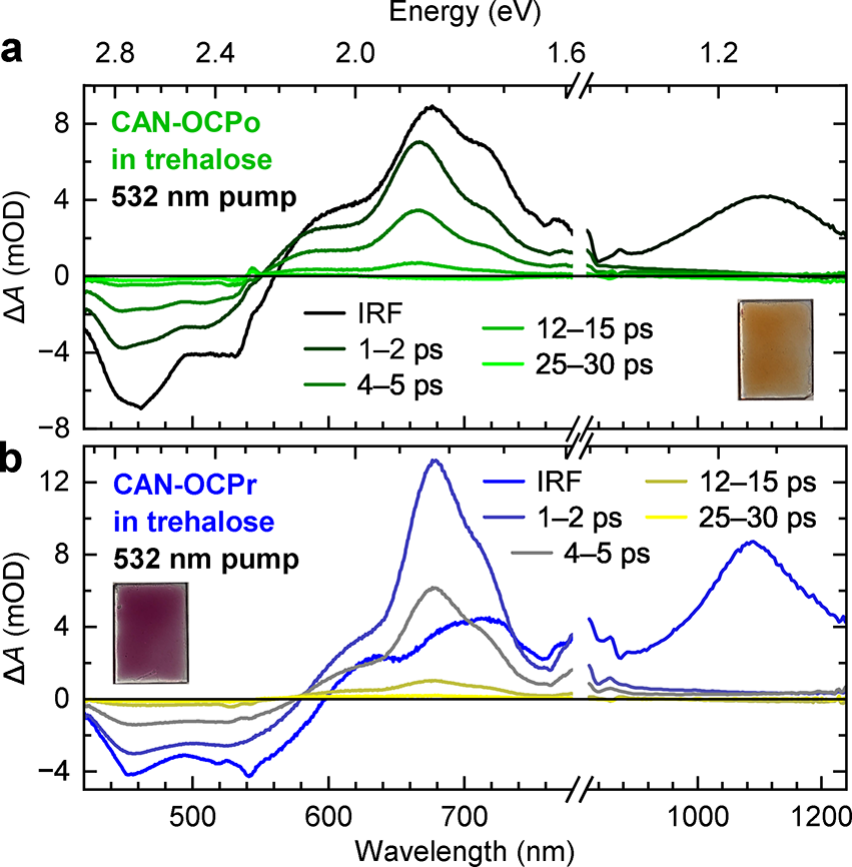
**Transient absorption spectra of CAN-binding OCPo (a) and OCPr (b) trapped in trehalose films.** The spectral time slices have been averaged between the times indicated in the figure and are consistent with an S_2_ → S_1_ → S_0_ decay scheme in both cases, with no discernible long-lived features (see also global lifetime analysis of the data in SI section S4.2). The films were excited with 532 nm, 5 kHz, ∼100 fs, and 200 μJ cm^–2^ pump pulses.

**Figure 4:**
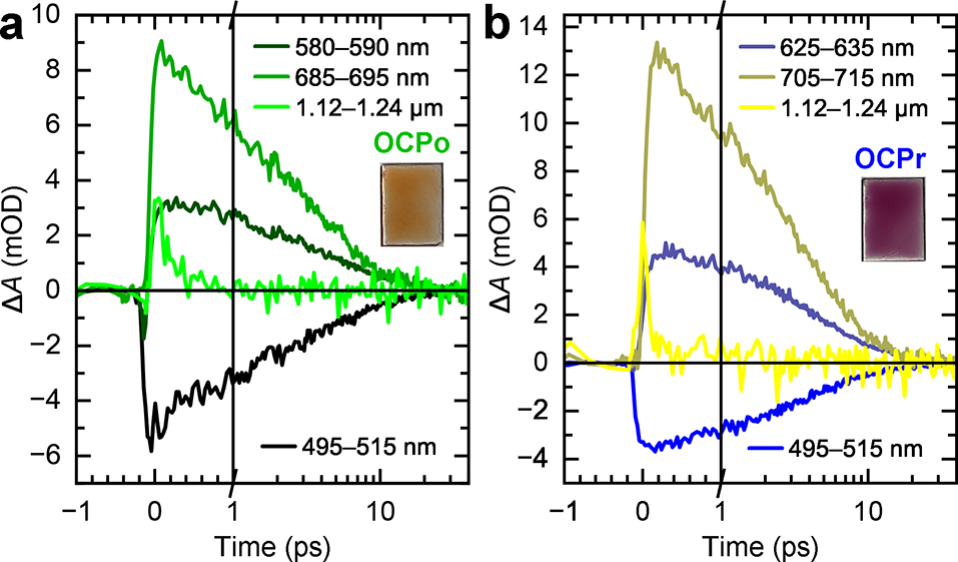
**Transient absorption dynamics of CAN-binding OCPo (a) and OCPr (b) in trehalose glass.** The dynamics have been averaged between the wavelengths indicated in the legend and demonstrate that no discernible long-lived features are seen. The films were excited with 532 nm, 5 kHz, ∼100 fs, and 200 μJ cm^–2^ pulses. Note that the plots have a linear time axis up to 1 ps and subsequently logarithmic up to 40 ps.

The lack of iSF in the protein-twisted carotenoid in OCPo appears to question the currently accepted hypothesis that the determinant for iSF in carotenoids is a twist along its backbone.^73^ Indeed, while the carotenoid environments in OCP and photosynthetic complexes are very different, the carotenoids in OCPo and LHCs seem to demonstrate similar backbone twists (see Figure S3 and the SI text for further comparison).^73^ Therefore, the lack of iSF in OCPo suggests that it would be worth revisiting the mechanism of SF in purple bacterial LHCs, particularly considering recent work on the nature of intermediate triplet-pair states involved in SF, as probed by magnetic-field-dependent measurements.^77,78^ We therefore return to the original studies of SF in these LHC systems and discuss them in light of this recent work.^77,78^

Singlet fission in LHCs was first observed in a series of experiments that probed their magnetic-field-dependent fluorescence.^79−81^ Representative data for oxidized cells from *Rhodobacter sphaeroides* 2.4.1 from ref (79) are reproduced in Figure 5; similar behavior has been reported for whole cells and isolated LHCs from several strains of purple bacteria.^79−81,84^ The shape of the magnetic field effect (MFE) in Figure 5, with an initial dip in fluorescence as the field increases from 0 to 40 mT and then a rise in fluorescence to saturation beyond 100 mT, is a characteristic signature of SF.

**Figure 5:**
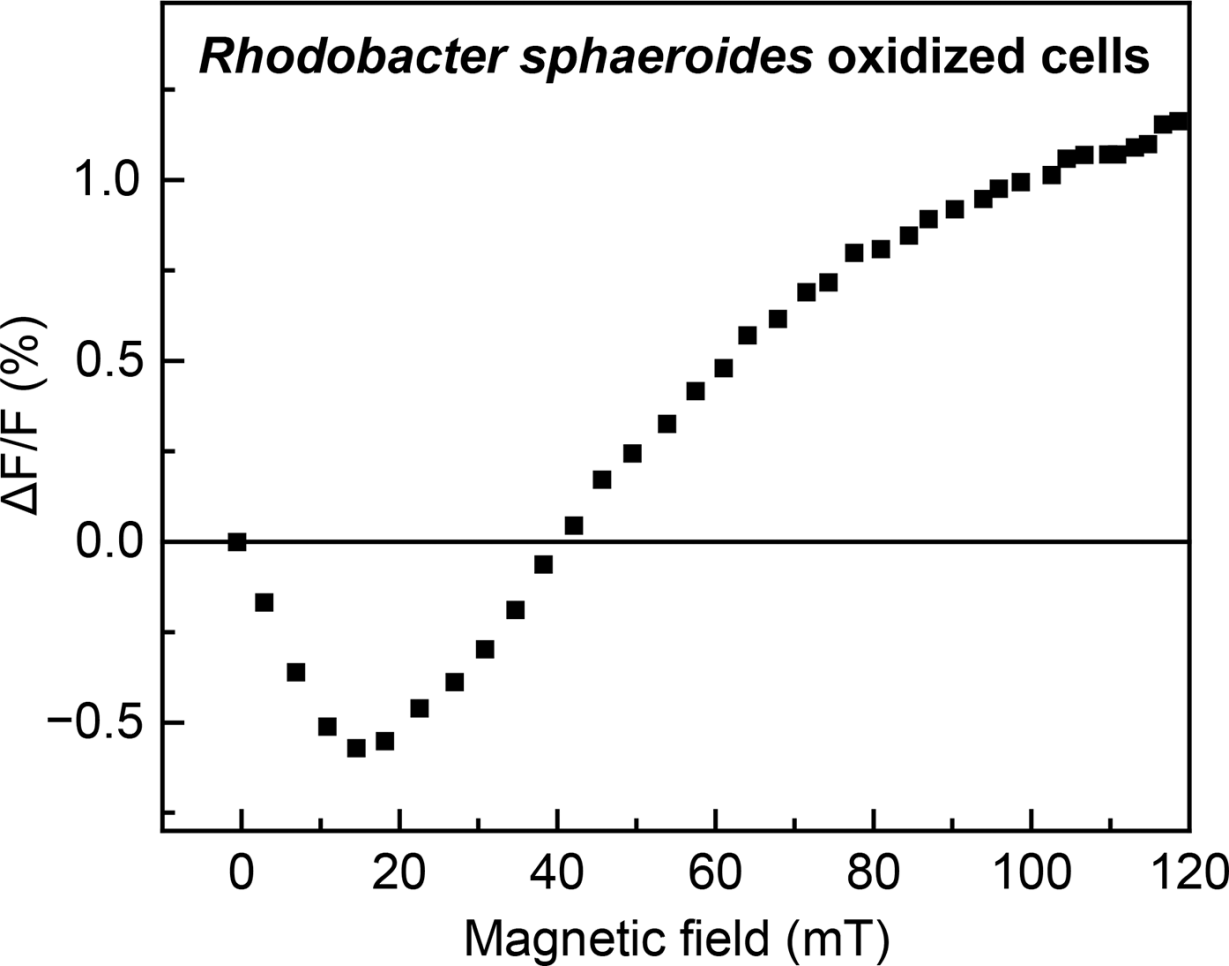
**Magnetic field effect (MFE) of *Rhodobacter sphaeroides***. Data were taken from ref (79). The data are plotted as the normalized change in fluorescence, Δ*F*/*F* (detected at 890 nm), as a function of the magnetic field strength upon 515 nm excitation in oxidized cells of *Rhodobacter sphaeroides* 2.4.1, with 1 mM K_3_Fe(CN)_6_ added. *A*_850–960_ = 0.35; optical path length = 2 mm.

This behavior is very well described by the kinetic model of SF by Johnson and Merrifield,^51,91−94^ published in the 1960s and 1970s. Recent work^77,78^ shows that this low-field Merrifield-type MFE behavior can only be observed when the intertriplet exchange interaction *J* is negligible or, more precisely, when *J* ≪ *D*,^95^ where *D* is the intratriplet dipolar zero-field splitting parameter. In carotenoids, and indeed most organic chromophores, *D* is relatively small, on the order of 4–10 μeV.^96,97^ If *J* increases beyond *D*, the MFE has a different behavior, showing dips in fluorescence at much higher field strengths.^77,78,98,99^ Therefore, to determine whether SF along a single carotenoid chain is capable of producing the measured MFEs in LHCs, we must estimate the values of *J* and *D*.

Before doing so, we make several observations about the carotenoids involved in SF in LHCs of purple bacteria: (1) the S_0_ → S_2_ absorbance spectra of the carotenoids in light-harvesting antenna are similar to those of their all-*trans* forms in organic solvent and depend sensitively on the carotenoid conjugation length.^74,100^ A full break in conjugation along the chain would lead to a dramatic blue shift of the carotenoid absorption feature that is not observed. (2) The carotenoid T_1_ → T_*n*_ excited-state absorption feature seen in transient absorption of LHCs^71,72,74,75^ is very similar to that seen in aggregated carotenoids of comparable conjugation lengths forming triplets by intermolecular SF.^65−67,70^ The T_1_ → T_*n*_ feature is also sensitive to the carotenoid conjugation length,^74,101,102^ and a conjugation break along the chain would similarly lead to a blue shift that is not observed. (3) The dipolar *D* and *E* parameters of the SF-generated triplets in LHCs from transient electron parametric resonance (EPR) spectroscopy are similar to full-chain triplet *D* and *E* parameters rather than to their half-chain alternatives.^84^ These observations suggest that the conjugation along the chain is not broken, even in the LHC antenna protein, and therefore that the triplets at either end of the chain maintain orbital overlap and, presumably, non-negligible *J*.

The exchange interaction, *J*, between triplets within a pair is equal to one-sixth of the energy difference between the pure singlet triplet pair, denoted ^1^(TT), and the pure quintet, ^5^(TT).^3^ In addition, to first approximation, the energy of ^5^(TT) is equal to twice the free triplet energy.^3,56,103^ In carotenoids, as described above, the lowest-energy singlet state (S_1_) is predominantly a pure singlet ^1^(TT) state. Therefore, a comparison between twice the energy of a triplet on half a chain against the energy of S_1_ on a full chain provides an indication of the exchange interaction.

Recent high-level density matrix renormalization group (DMRG) calculations of the Pariser–Parr–Pople–Peierls Hamiltonian^56^ show that 2 × *E*(T_1_) for a half chain is higher in energy than S_1_ (≈^1^(TT)) for a full chain at all conjugation lengths. This is supported by experimentally determined energies: for diphenylexatriene with *N* = 5 conjugated double bonds, 2 × *E*(T_1_) = 3.02 ± 0.1 eV,^101,104^ while for spheroidene with twice the number of double bonds (*N* = 10), *E*(S_1_) = 1.77 eV.^105^ This would indicate an exchange interaction of *J* = 0.2 eV, which is orders of magnitude larger than the dipolar parameter *D* ∼ 4–10 μeV.^96,97^ These energies indicate that the triplets within ^1^(TT) should be strongly exchange-coupled.

The triplets within a single carotenoid chain are therefore exchange-coupled (*J* ≫ *D*) even in a protein that twists the carotenoid backbone,^73^ as no breaks in conjugation along the carotenoid chain have been observed (i.e., no observable shifts in absorption spectra^74^ or changes in dipolar *D* and *E* parameters^84^). Therefore, MFEs such as those reproduced in Figure 5, that were the initial proof of SF in purple bacteria, cannot be explained with an intramolecular model of SF. We speculate that the mechanism for singlet fission in light-harvesting complexes must be intermolecular, possibly occurring via the neighboring bacteriochlorophyll *a* molecules in the protein complex.

We conclude that singlet fission (SF) to produce long-lived triplets does not occur along a single twisted carotenoid chain in the OCP and is unlikely to occur on a single carotenoid chain in purple bacterial light-harvesting complexes (LHCs), contrary to the current notion.^71−75^ We conclude this because (1) immobilized OCPo—an uncomplicated, minimal carotenoprotein—shows similar twisted carotenoid geometry to LHCs but shows no evidence of SF and (2) the MFEs that identified SF in purple bacteria are irreconcilable with iSF without a significant break in conjugation (which is not observed in LHCs). These findings therefore call into question the mechanism of SF observed in LHCs.

## Data Availability

Data are available on the University of Sheffield’s repository, ORDA.

## Supporting Information Available

Experimental methods, including canthaxanthin-binding OCP production, OCPo and OCPr in sugar glasses preparation, spectroscopic setups, and data analysis procedures; back-conversion of solution OCPr to OCPo in the dark; comparison of the resonance Raman spectra of OCPo and OCPr of this work against previously published spectra of purple bacterial LHCs; resonance Raman spectrum of blank trehalose; transient absorption pump spectrum; results of global lifetime analysis on the transient absorption data (PDF)

## References

[ref1] Bardeen C. J. The Structure and Dynamics of Molecular Excitons. Annu. Rev. Phys. Chem. 2014, 65, 127–148. 10.1146/annurev-physchem-040513-103654. 24313684

[ref2] Smith M. B.; Michl J. Recent Advances in Singlet Fission. Annu. Rev. Phys. Chem. 2013, 64, 361–386. 10.1146/annurev-physchem-040412-110130. 23298243

[ref3] Musser A. J.; Clark J. Triplet-Pair States in Organic Semiconductors. Annu. Rev. Phys. Chem. 2019, 70, 323–351. 10.1146/annurev-physchem-042018-052435. 31174458

[ref4] Ullrich T.; Munz D.; Guldi D. M. Unconventional Singlet Fission Materials. Chem. Soc. Rev. 2021, 50, 3485–3518. 10.1039/D0CS01433H. 33496309

[ref5] Kim H.; Zimmerman P. M. Coupled Double Triplet State in Singlet Fission. Phys. Chem. Chem. Phys. 2018, 20, 30083–30094. 10.1039/C8CP06256K. 30484452

[ref6] Hanna M. C.; Nozik A. J. Solar Conversion Efficiency of Photovoltaic and Photoelectrolysis Cells with Carrier Multiplication Absorbers. J. Appl. Phys. 2006, 100, 074510 10.1063/1.2356795.

[ref7] Rao A.; Friend R. H. Harnessing Singlet Exciton Fission to Break the Shockley–Queisser Limit. Nat. Rev. Mater. 2017, 2, 17063 10.1038/natrevmats.2017.63.

[ref8] Ehrler B.; Yanai N.; Nienhaus L. Up- and Down-Conversion in Molecules and Materials. J. Chem. Phys. 2021, 154, 070401 10.1063/5.0045323. 33607873

[ref9] Daiber B.; van den Hoven K.; Futscher M. H.; Ehrler B. Realistic Efficiency Limits for Singlet-Fission Silicon Solar Cells. ACS Energy Lett. 2021, 6, 2800–2808. 10.1021/acsenergylett.1c00972. 34476299 PMC8389984

[ref10] Ehrler B.; Ho-Baillie A. W. Y.; Hutter E. M.; Milić J. V.; Tayebjee M. J. Y.; Wilson M. W. B. Scalable Ways to Break the Efficiency Limit of Single-Junction Solar Cells. Appl. Phys. Lett. 2022, 120, 010402 10.1063/5.0081049.

[ref11] Liu Y.; Zhang C.; Wang R.; Zhang B.; Tan Z.; Wang X.; Xiao M. Large Optical Nonlinearity Induced by Singlet Fission in Pentacene Films. Angew. Chem., Int. Ed. 2015, 54, 6222–6226. 10.1002/anie.201501396. 25845461

[ref12] Zhao M.; Liu K.; Zhang Y.-D.; Wang Q.; Li Z.-G.; Song Y.-L.; Zhang H.-L. Singlet Fission Induced Giant Optical Limiting Responses of Pentacene Derivatives. Mater. Horiz. 2015, 2, 619–624. 10.1039/C5MH00120J.

[ref13] Tonami T.; Nagami T.; Okada K.; Yoshida W.; Nakano M. Singlet-Fission-Induced Enhancement of Third-Order Nonlinear Optical Properties of Pentacene Dimers. ACS Omega 2019, 4, 16181–16190. 10.1021/acsomega.9b02378. 31592485 PMC6777123

[ref14] Nagata R.; Nakanotani H.; Potscavage W. J.; Adachi C. Exploiting Singlet Fission in Organic Light-Emitting Diodes. Adv. Mater. 2018, 30, 1801484 10.1002/adma.201801484. 29974520

[ref15] Teichen P. E.; Eaves J. D. Collective Aspects of Singlet Fission in Molecular Crystals. J. Chem. Phys. 2015, 143, 044118 10.1063/1.4922644. 26233118

[ref16] Bardeen C. J. Time Dependent Correlations of Entangled States with Nondegenerate Branches and Possible Experimental Realization Using Singlet Fission. J. Chem. Phys. 2019, 151, 124503 10.1063/1.5117155. 31575193

[ref17] Marcus M.; Barford W. Triplet-Triplet Decoherence in Singlet Fission. Phys. Rev. B 2020, 102, 035134 10.1103/PhysRevB.102.035134.

[ref18] Smyser K. E.; Eaves J. D. Singlet Fission for Quantum Information and Quantum Computing: The Parallel JDE Model. Sci. Rep. 2020, 10, 18480 10.1038/s41598-020-75459-x. 33116218 PMC7595132

[ref19] Einzinger M.; Wu T.; Kompalla J. F.; Smith H. L.; Perkinson C. F.; Nienhaus L.; Wieghold S.; Congreve D. N.; Kahn A.; Bawendi M. G.; Baldo M. A. Sensitization of Silicon by Singlet Exciton Fission in Tetracene. Nature 2019, 571, 90–94. 10.1038/s41586-019-1339-4. 31270480

[ref20] Daiber B.; Maiti S.; Ferro S. M.; Bodin J.; van den Boom A. F. J.; Luxembourg S. L.; Kinge S.; Pujari S. P.; Zuilhof H.; Siebbeles L. D. A.; Ehrler B. Change in Tetracene Polymorphism Facilitates Triplet Transfer in Singlet Fission-Sensitized Silicon Solar Cells. J. Phys. Chem. Lett. 2020, 11, 8703–8709. 10.1021/acs.jpclett.0c02163. 32959663 PMC7569671

[ref21] Tavan P.; Schulten K. Electronic Excitations in Finite and Infinite Polyenes. Phys. Rev. B 1987, 36, 4337–4358. 10.1103/PhysRevB.36.4337. 9943414

[ref22] Schmidt M.; Tavan P. Electronic Excitations in Long Polyenes Revisited. J. Chem. Phys. 2012, 136, 124309 10.1063/1.3696880. 22462859

[ref23] Kraabel B.; Hulin D.; Aslangul C.; Lapersonne-Meyer C.; Schott M. Triplet Exciton Generation, Transport and Relaxation in Isolated Polydiacetylene Chains: Subpicosecond Pump-Probe Experiments. Chem. Phys. 1998, 227, 83–98. 10.1016/S0301-0104(97)00200-0.

[ref24] Pandya R.; Gu Q.; Cheminal A.; Chen R. Y.; Booker E. P.; Soucek R.; Schott M.; Legrand L.; Mathevet F.; Greenham N. C.; Barisien T.; Musser A. J.; Chin A. W.; Rao A. Optical Projection and Spatial Separation of Spin-Entangled Triplet Pairs from the S_1_ (2^1^ A_g_^–^) State of Pi-Conjugated Systems. Chem 2020, 6, 2826–2851. 10.1016/j.chempr.2020.09.011.

[ref25] Lanzani G.; Cerullo G.; Zavelani-Rossi M.; De Silvestri S.; Comoretto D.; Musso G.; Dellepiane G. Triplet-Exciton Generation Mechanism in a New Soluble (Red-Phase) Polydiacetylene. Phys. Rev. Lett. 2001, 87, 187402 10.1103/PhysRevLett.87.187402.

[ref26] Musser A. J.; Al-Hashimi M.; Maiuri M.; Brida D.; Heeney M.; Cerullo G.; Friend R. H.; Clark J. Activated Singlet Exciton Fission in a Semiconducting Polymer. J. Am. Chem. Soc. 2013, 135, 12747–12754. 10.1021/ja405427j. 23883167

[ref27] Lafalce E.; Jiang X.; Zhang C. Generation and Recombination Kinetics of Optical Excitations in Poly(3-dodecylthienylenevinylene) with Controlled Regioregularity. J. Phys. Chem. B 2011, 115, 13139–13148. 10.1021/jp2066666. 21916464

[ref28] Musser A. J.; Al-Hashimi M.; Heeney M.; Clark J. Heavy-Atom Effects on Intramolecular Singlet Fission in a Conjugated Polymer. J. Chem. Phys. 2019, 151, 044902 10.1063/1.5110269. 31370564

[ref29] Busby E.; Xia J.; Wu Q.; Low J. Z.; Song R.; Miller J. R.; Zhu X.-Y.; Campos L. M.; Sfeir M. Y. A Design Strategy for Intramolecular Singlet Fission Mediated by Charge-Transfer States in Donor-Acceptor Organic Materials. Nat. Mater. 2015, 14, 426–433. 10.1038/nmat4175. 25581625

[ref30] Kasai Y.; Tamai Y.; Ohkita H.; Benten H.; Ito S. Ultrafast Singlet Fission in a Push–Pull Low-Bandgap Polymer Film. J. Am. Chem. Soc. 2015, 137, 15980–15983. 10.1021/jacs.5b09361. 26654295

[ref31] Fallon K. J.; et al. Exploiting Excited-State Aromaticity To Design Highly Stable Singlet Fission Materials. J. Am. Chem. Soc. 2019, 141, 13867–13876. 10.1021/jacs.9b06346. 31381323

[ref32] Huynh U. N. V.; Basel T. P.; Ehrenfreund E.; Li G.; Yang Y.; Mazumdar S.; Vardeny Z. V. Transient Magnetophotoinduced Absorption Studies of Photoexcitations in π-Conjugated Donor-Acceptor Copolymers. Phys. Rev. Lett. 2017, 119, 017401 10.1103/PhysRevLett.119.017401. 28731770

[ref33] Casado J.; Ponce Ortiz R.; López Navarrete J. T. Quinoidal Oligothiophenes: New Properties Behind an Unconventional Electronic Structure. Chem. Soc. Rev. 2012, 41, 5672–5686. 10.1039/c2cs35079c. 22806576

[ref34] Varnavski O.; Abeyasinghe N.; Aragó J.; Serrano-Pérez J. J.; Ortí E.; López Navarrete J. T.; Takimiya K.; Casanova D.; Casado J.; Goodson T. High Yield Ultrafast Intramolecular Singlet Exciton Fission in a Quinoidal Bithiophene. J. Phys. Chem. Lett. 2015, 6, 1375–1384. 10.1021/acs.jpclett.5b00198. 26263138

[ref35] Chien A. D.; Molina A. R.; Abeyasinghe N.; Varnavski O. P.; Goodson T.; Zimmerman P. M. Structure and Dynamics of the ^1^(TT) State in a Quinoidal Bithiophene: Characterizing a Promising Intramolecular Singlet Fission Candidate. J. Phys. Chem. C 2015, 119, 28258–28268. 10.1021/acs.jpcc.5b07786.

[ref36] Kim H.; Keller B.; Ho-Wu R.; Abeyasinghe N.; Vázquez R. J.; Goodson T.; Zimmerman P. M. Enacting Two-Electron Transfer from a Double-Triplet State of Intramolecular Singlet Fission. J. Am. Chem. Soc. 2018, 140, 7760–7763. 10.1021/jacs.8b01884. 29741376

[ref37] Kawata S.; Pu Y.-J.; Saito A.; Kurashige Y.; Beppu T.; Katagiri H.; Hada M.; Kido J. Singlet Fission of Non-Polycyclic Aromatic Molecules in Organic Photovoltaics. Adv. Mater. 2016, 28, 1585–1590. 10.1002/adma.201504281. 26663207

[ref38] Ullrich T.; Pinter P.; Messelberger J.; Haines P.; Kaur R.; Hansmann M. M.; Munz D.; Guldi D. M. Singlet Fission in Carbene-Derived Diradicaloids. Angew. Chem., Int. Ed. 2020, 59, 7906–7914. 10.1002/anie.202001286. PMC7317569 32129920

[ref39] Wu Y.; Wang Y.; Chen J.; Zhang G.; Yao J.; Zhang D.; Fu H. Intramolecular Singlet Fission in an Antiaromatic Polycyclic Hydrocarbon. Angew. Chem., Int. Ed. 2017, 56, 9400–9404. 10.1002/anie.201704668. 28626959

[ref40] Liu Y.; Wu Y.; Wang L.; Wang L.; Yao J.; Fu H. Efficient Triplet Pair Separation from Intramolecular Singlet Fission in Dibenzopentalene Derivatives. Sci. China: Chem. 2019, 62, 1037–1043. 10.1007/s11426-019-9482-y.

[ref41] Canniffe D. P.; Hitchcock A. In Encyclopedia of Biological Chemistry III, 3rd ed.; Jez J., Ed.; Elsevier: Oxford, U.K., 2021; Vol. 2; pp 163–185.

[ref42] Yabuzaki J. Carotenoids Database: Structures, Chemical Fingerprints and Distribution among Organisms. Database 2017, 2017, bax004 10.1093/database/bax004. 28365725 PMC5574413

[ref43] Leverenz R. L.; Sutter M.; Wilson A.; Gupta S.; Thurotte A.; Bourcier de Carbon C.; Petzold C. J.; Ralston C.; Perreau F.; Kirilovsky D.; Kerfeld C. A. A 12 Å Carotenoid Translocation in a Photoswitch Associated with Cyanobacterial Photoprotection. Science 2015, 348, 1463–1466. 10.1126/science.aaa7234. 26113721

[ref44] Kerfeld C. A.; Sutter M.; Leverenz R. L. 4XB5: Structure of Orange Carotenoid Protein Binding Canthaxanthin. Protein Data Bank, 2014. 10.2210/pdb4XB5/pdb.

[ref45] Kerfeld C. A.; Sutter M.; Leverenz R. L. 4XB4: Structure of the N-Terminal Domain of OCP Binding Canthaxanthin. Protein Data Bank, 2014. 10.2210/pdb4XB4/pdb.

[ref46] Yong C. K.; et al. The Entangled Triplet Pair State in Acene and Heteroacene Materials. Nat. Commun. 2017, 8, 15953 10.1038/ncomms15953. 28699637 PMC5510179

[ref47] Pun A. B.; Asadpoordarvish A.; Kumarasamy E.; Tayebjee M. J. Y.; Niesner D.; McCamey D. R.; Sanders S. N.; Campos L. M.; Sfeir M. Y. Ultra-Fast Intramolecular Singlet Fission to Persistent Multiexcitons by Molecular Design. Nat. Chem. 2019, 11, 821–828. 10.1038/s41557-019-0297-7. 31406323

[ref48] Korovina N. V.; Chang C. H.; Johnson J. C. Spatial Separation of Triplet Excitons Drives Endothermic Singlet Fission. Nat. Chem. 2020, 12, 391–398. 10.1038/s41557-020-0422-7. 32123340

[ref49] Wang Z.; Liu H.; Xie X.; Zhang C.; Wang R.; Chen L.; Xu Y.; Ma H.; Fang W.; Yao Y.; Sang H.; Wang X.; Li X.; Xiao M. Free-Triplet Generation with Improved Efficiency in Tetracene Oligomers Through Spatially Separated Triplet Pair States. Nat. Chem. 2021, 13, 559–567. 10.1038/s41557-021-00665-7. 33833447

[ref50] Wilson M. W. B.; Rao A.; Clark J.; Kumar R. S. S.; Brida D.; Cerullo G.; Friend R. H. Ultrafast Dynamics of Exciton Fission in Polycrystalline Pentacene. J. Am. Chem. Soc. 2011, 133, 11830–11833. 10.1021/ja201688h. 21755937

[ref51] Bossanyi D. G.; Matthiesen M.; Wang S.; Smith J. A.; Kilbride R. C.; Shipp J. D.; Chekulaev D.; Holland E.; Anthony J. E.; Zaumseil J.; Musser A. J.; Clark J. Emissive Spin-0 Triplet-Pairs Are a Direct Product of Triplet–Triplet Annihilation in Pentacene Single Crystals and Anthradithiophene Films. Nat. Chem. 2021, 13, 163–171. 10.1038/s41557-020-00593-y. 33288892

[ref52] Burdett J. J.; Piland G. B.; Bardeen C. J. Magnetic Field Effects and the Role of Spin States in Singlet Fission. Chem. Phys. Lett. 2013, 585, 1–10. 10.1016/j.cplett.2013.08.036.

[ref53] Piland G. B.; Bardeen C. J. How Morphology Affects Singlet Fission in Crystalline Tetracene. J. Phys. Chem. Lett. 2015, 6, 1841–1846. 10.1021/acs.jpclett.5b00569. 26263258

[ref54] Tayebjee M. J. Y.; Clady R. G. C. R.; Schmidt T. W. The Exciton Dynamics in Tetracene Thin Films. Phys. Chem. Chem. Phys. 2013, 15, 14797–14805. 10.1039/c3cp52609g. 23907164

[ref55] Wilson M. W. B.; Rao A.; Johnson K.; Gélinas S.; di Pietro R.; Clark J.; Friend R. H. Temperature-Independent Singlet Exciton Fission in Tetracene. J. Am. Chem. Soc. 2013, 135, 16680–16688. 10.1021/ja408854u. 24148017

[ref56] Valentine D. J.; Manawadu D.; Barford W. Higher-Energy Triplet-Pair States in Polyenes and Their Role in Intramolecular Singlet Fission. Phys. Rev. B 2020, 102, 125107 10.1103/PhysRevB.102.125107.

[ref57] Manawadu D.; Valentine D. J.; Marcus M.; Barford W. Singlet Triplet-Pair Production and Possible Singlet-Fission in Carotenoids. J. Phys. Chem. Lett. 2022, 13, 1344–1349. 10.1021/acs.jpclett.1c03812. 35108016 PMC9084603

[ref58] Barford W. Theory of the Dark State of Polyenes and Carotenoids. Phys. Rev. B 2022, 106, 035201 10.1103/PhysRevB.106.035201.

[ref59] Balevičius V.; Abramavicius D.; Polívka T.; Galestian Pour A.; Hauer J. A Unified Picture of S*in Carotenoids. J. Phys. Chem. Lett. 2016, 7, 3347–3352. 10.1021/acs.jpclett.6b01455. 27509302 PMC5011297

[ref60] Balevičius V.; Wei T.; Di Tommaso D.; Abramavicius D.; Hauer J.; Polívka T.; Duffy C. D. P. The Full Dynamics of Energy Relaxation in Large Organic Molecules: From Photo-Excitation to Solvent Heating. Chem. Sci. 2019, 10, 4792–4804. 10.1039/C9SC00410F. 31183032 PMC6521204

[ref61] Taffet E. J.; Fassioli F.; Toa Z. S. D.; Beljonne D.; Scholes G. D. Uncovering Dark Multichromophoric States in Peridinin–Chlorophyll–Protein. J. R. Soc., Interface. 2020, 17, 20190736 10.1098/rsif.2019.0736. 32183641 PMC7115236

[ref62] Barford W.; Bursill R. J.; Lavrentiev M. Y. Density-Matrix Renormalization-Group Calculations of Excited States of Linear Polyenes. Phys. Rev. B 2001, 63, 195108 10.1103/PhysRevB.63.195108.

[ref63] Polak D. W.; Musser A. J.; Sutherland G. A.; Auty A.; Branchi F.; Dzurnak B.; Chidgey J.; Cerullo G.; Hunter C. N.; Clark J. Band-Edge Excitation of Carotenoids Removes S* Revealing Triplet-Pair Contributions to the S_1_ Absorption Spectrum. arXiv (Physics.Chemical Physics), January 15, 2019, 1901.04900, ver. 1. https://arxiv.org/abs/1901.04900 (accessed 2023-04-26).

[ref64] Yablon L. M.; Sanders S. N.; Miyazaki K.; Kumarasamy E.; He G.; Choi B.; Ananth N.; Sfeir M. Y.; Campos L. M. Singlet Fission and Triplet Pair Recombination in Bipentacenes with a Twist. Mater. Horiz. 2022, 9, 462–470. 10.1039/D1MH01201K. 34846410

[ref65] Musser A. J.; Maiuri M.; Brida D.; Cerullo G.; Friend R. H.; Clark J. The Nature of Singlet Exciton Fission in Carotenoid Aggregates. J. Am. Chem. Soc. 2015, 137, 5130–5139. 10.1021/jacs.5b01130. 25825939 PMC4440407

[ref66] Zhang D.; Tan L.; Dong J.; Yi J.; Wang P.; Zhang J. Structure and Excitation Dynamics of β-Carotene Aggregates in Cetyltrimethylammonium Bromide Micelle. Chem. Res. Chin. Univ. 2018, 34, 643–648. 10.1007/s40242-018-7379-8.

[ref67] Chang H.-T.; Chang Y.-Q.; Han R.-M.; Wang P.; Zhang J.-P.; Skibsted L. H. Singlet Fission Reaction of Light-Exposed β-Carotene Bound to Bovine Serum Albumin. A Novel Mechanism in Protection of Light-Exposed Tissue by Dietary Carotenoids. J. Agric. Food Chem. 2017, 65, 6058–6062. 10.1021/acs.jafc.7b01616. 28669184

[ref68] Wang C.; Tauber M. J. High-Yield Singlet Fission in a Zeaxanthin Aggregate Observed by Picosecond Resonance Raman Spectroscopy. J. Am. Chem. Soc. 2010, 132, 13988–13991. 10.1021/ja102851m. 20857932

[ref69] Wang C.; Schlamadinger D. E.; Desai V.; Tauber M. J. Triplet Excitons of Carotenoids Formed by Singlet Fission in a Membrane. ChemPhysChem 2011, 12, 2891–2894. 10.1002/cphc.201100571. 21910205

[ref70] Sutherland G. A.; Polak D.; Swainsbury D. J. K.; Wang S.; Spano F. C.; Auman D. B.; Bossanyi D. G.; Pidgeon J. P.; Hitchcock A.; Musser A. J.; Anthony J. E.; Dutton P. L.; Clark J.; Hunter C. N. A Thermostable Protein Matrix for Spectroscopic Analysis of Organic Semiconductors. J. Am. Chem. Soc. 2020, 142, 13898–13907. 10.1021/jacs.0c05477. 32672948

[ref71] Gradinaru C. C.; Kennis J. T. M.; Papagiannakis E.; van Stokkum I. H. M.; Cogdell R. J.; Fleming G. R.; Niederman R. A.; van Grondelle R. An Unusual Pathway of Excitation Energy Deactivation in Carotenoids: Singlet-to-Triplet Conversion on an Ultrafast Timescale in a Photosynthetic Antenna. Proc. Natl. Acad. Sci. U.S.A. 2001, 98, 2364–2369. 10.1073/pnas.051501298. 11226245 PMC30144

[ref72] Papagiannakis E.; Kennis J. T. M.; van Stokkum I. H. M.; Cogdell R. J.; van Grondelle R. An Alternative Carotenoid-to-Bacteriochlorophyll Energy Transfer Pathway in Photosynthetic Light Harvesting. Proc. Natl. Acad. Sci. U.S.A. 2002, 99, 6017–6022. 10.1073/pnas.092626599. 11972067 PMC122894

[ref73] Yu J.; Fu L.-M.; Yu L.-J.; Shi Y.; Wang P.; Wang-Otomo Z.-Y.; Zhang J.-P. Carotenoid Singlet Fission Reactions in Bacterial Light Harvesting Complexes As Revealed by Triplet Excitation Profiles. J. Am. Chem. Soc. 2017, 139, 15984–15993. 10.1021/jacs.7b09809. 29053262

[ref74] Niedzwiedzki D. M.; Swainsbury D. J. K.; Martin E. C.; Hunter C. N.; Blankenship R. E. Origin of the S* Excited State Feature of Carotenoids in Light-Harvesting Complex 1 from Purple Photosynthetic Bacteria. J. Phys. Chem. B 2017, 121, 7571–7585. 10.1021/acs.jpcb.7b04251. 28719215

[ref75] Zhang Y.; Qi C.-H.; Yamano N.; Wang P.; Yu L.-J.; Wang-Otomo Z.-Y.; Zhang J.-P. Carotenoid Single-Molecular Singlet Fission and the Photoprotection of a Bacteriochlorophyll b-Type Core Light-Harvesting Antenna. J. Phys. Chem. Lett. 2022, 13, 3534–3541. 10.1021/acs.jpclett.2c00519. 35420425

[ref76] Kish E.; Pinto M. M. M.; Kirilovsky D.; Spezia R.; Robert B. Echinenone Vibrational Properties: From Solvents to the Orange Carotenoid Protein. Biochim. Biophys. Acta, Bioenerg. 2015, 1847, 1044–1054. 10.1016/j.bbabio.2015.05.010. 26003409

[ref77] Bayliss S. L.; Weiss L. R.; Rao A.; Friend R. H.; Chepelianskii A. D.; Greenham N. C. Spin Signatures of Exchange-Coupled Triplet Pairs Formed by Singlet Fission. Phys. Rev. B 2016, 94, 045204 10.1103/PhysRevB.94.045204.

[ref78] Bossanyi D. G.; Sasaki Y.; Wang S.; Chekulaev D.; Kimizuka N.; Yanai N.; Clark J. Spin Statistics for Triplet–Triplet Annihilation Upconversion: Exchange Coupling, Intermolecular Orientation, and Reverse Intersystem Crossing. JACS Au 2021, 1, 2188–2201. 10.1021/jacsau.1c00322. 34977890 PMC8715495

[ref79] Kingma H.; van Grondelle R.; Duysens L. Magnetic-Field Effects in Photosynthetic Bacteria. I. Magnetic-Field-Induced Bacteriochlorophyll Emission Changes in the Reaction Center and the Antenna of Rhodospirillum rubrum, Rhodopseudomonas sphaeroides and Prosthecochloris aestuarii. Biochim. Biophys. Acta, Bioenerg. 1985, 808, 363–382. 10.1016/0005-2728(85)90146-X.

[ref80] Kingma H.; van Grondelle R.; Duysens L. Magnetic-Field Effects in Photosynthetic Bacteria. II. Formation of Triplet States in the Reaction Center and the Antenna of *Rhodospirillum rubrum and Rhodopseudomonas sphaeroides*. Magnetic-Field Effects. Biochim. Biophys. Acta, Bioenerg. 1985, 808, 383–399. 10.1016/0005-2728(85)90147-1.

[ref81] Rademaker H.; Hoff A. J.; Van Grondelle R.; Duysens L. N. Carotenoid Triplet Yields in Normal and Deuterated *Rhodospirillum rubrum*. Biochim. Biophys. Acta, Bioenerg. 1980, 592, 240–257. 10.1016/0005-2728(80)90185-1. 6773564

[ref82] Klenina I. B.; Makhneva Z. K.; Moskalenko A. A.; Kuzmin A. N.; Proskuryakov I. I. Singlet-Triplet Excitation Fission in Light-Harvesting Complexes of Photosynthetic Bacteria and in Isolated Carotenoids. Biophysics 2013, 58, 43–50. 10.1134/S0006350913010077. 23650855

[ref83] Klenina I. B.; Makhneva Z. K.; Moskalenko A. A.; Gudkov N. D.; Bolshakov M. A.; Pavlova E. A.; Proskuryakov I. I. Singlet-Triplet Fission of Carotenoid Excitation in Light-Harvesting LH2 Complexes of Purple Phototrophic Bacteria. Biochemistry (Moscow) 2014, 79, 235–241. 10.1134/S0006297914030092. 24821450

[ref84] Gryaznov A. A.; Klenina I. B.; Makhneva Z. K.; Moskalenko A. A.; Proskuryakov I. I. The Singlet–Triplet Fission of Carotenoid Excitation in Light-Harvesting Complexes from *Thermochromatium tepidum*. Biophysics 2019, 64, 847–852. 10.1134/S0006350919060083. 31201634

[ref85] Cunningham F. X.; Gantt E. A Portfolio of Plasmids for Identification and Analysis of Carotenoid Pathway Enzymes: *Adonis aestivalis* as a Case Study. Photosynth. Res. 2007, 92, 245–259. 10.1007/s11120-007-9210-0. 17634749

[ref86] Wilson A.; Punginelli C.; Gall A.; Bonetti C.; Alexandre M.; Routaboul J.-M.; Kerfeld C. A.; van Grondelle R.; Robert B.; Kennis J. T. M.; Kirilovsky D. A Photoactive Carotenoid Protein Acting as Light Intensity Sensor. Proc. Natl. Acad. Sci. U.S.A. 2008, 105, 12075–12080. 10.1073/pnas.0804636105. 18687902 PMC2575289

[ref87] Niedzwiedzki D. M.; Liu H.; Blankenship R. E. Excited State Properties of 3′-Hydroxyechinenone in Solvents and in the Orange Carotenoid Protein from *Synechocystis* sp. PCC 6803. J. Phys. Chem. B 2014, 118, 6141–6149. 10.1021/jp5041794. 24846130

[ref88] Bondanza M.; Cupellini L.; Faccioli P.; Mennucci B. Molecular Mechanisms of Activation in the Orange Carotenoid Protein Revealed by Molecular Dynamics. J. Am. Chem. Soc. 2020, 142, 21829–21841. 10.1021/jacs.0c10461. 33332967 PMC7775743

[ref89] Kurashov V.; Gorka M.; Milanovsky G. E.; Johnson T. W.; Cherepanov D. A.; Semenov A. Y.; Golbeck J. H. Critical Evaluation of Electron Transfer Kinetics in P700–FA/FB, P700–FX, and P700–A1 Photosystem I Core Complexes in Liquid and in Trehalose Glass. Biochim. Biophys. Acta, Bioenerg. 2018, 1859, 1288–1301. 10.1016/j.bbabio.2018.09.367. 30463673

[ref90] Saito S.; Tasumi M. Normal-Coordinate Analysis of Retinal Isomers and Assignments of Raman and Infrared Bands. J. Raman Spectrosc. 1983, 14, 236–245. 10.1002/jrs.1250140405.

[ref91] Merrifield R. E. Diffusion and Mutual Annihilation of Triplet Excitons in Organic Crystals. Acc. Chem. Res. 1968, 1, 129–135. 10.1021/ar50005a001.

[ref92] Groff R. P.; Avakian P.; Merrifield R. E. Coexistence of Exciton Fission and Fusion in Tetracene Crystals. Phys. Rev. B 1970, 1, 815–817. 10.1103/PhysRevB.1.815.

[ref93] Piland G. B.; Burdett J. J.; Kurunthu D.; Bardeen C. J. Magnetic Field Effects on Singlet Fission and Fluorescence Decay Dynamics in Amorphous Rubrene. J. Phys. Chem. C 2013, 117, 1224–1236. 10.1021/jp309286v.

[ref94] Tapping P. C.; Huang D. M. Comment on “Magnetic Field Effects on Singlet Fission and Fluorescence Decay Dynamics in Amorphous Rubrene. J. Phys. Chem. C 2016, 120, 25151–25157. 10.1021/acs.jpcc.6b04934.

[ref95] Benk H.; Sixl H. Theory of Two Coupled Triplet States. Mol. Phys. 1981, 42, 779–801. 10.1080/00268978100100631.

[ref96] Frick J.; Schütz J. U. V.; Wolf H. C.; Kothe G. First Detection of the (Nonphosphorescent) Triplet State in Single Crystals of β-Carotene. Mol. Cryst. Liq. Cryst. 1990, 183, 269–272. 10.1080/15421409008047463.

[ref97] Teki Y.; von Schütz J.; Wachtel H.; Weiss V.; Wolf H. Triplet Excitons in Diphenylbutadiene and Diphenylhexatriene Single Crystals by Zero-Field Delayed Fluorescence ODMR. Chem. Phys. Lett. 1994, 225, 124–130. 10.1016/0009-2614(94)00649-0.

[ref98] Bayliss S. L.; et al. Site-Selective Measurement of Coupled Spin Pairs in an Organic Semiconductor. Proc. Natl. Acad. Sci. U.S.A. 2018, 115, 5077–5082. 10.1073/pnas.1718868115. 29720443 PMC5960292

[ref99] Ishikawa K.; Yago T.; Wakasa M. Exploring the Structure of an Exchange-Coupled Triplet Pair Generated by Singlet Fission in Crystalline Diphenylhexatriene: Anisotropic Magnetic Field Effects on Fluorescence in High Fields. J. Phys. Chem. C 2018, 122, 22264–22272. 10.1021/acs.jpcc.8b06026.

[ref100] Chynwat V.; Frank H. A. The Application of the Energy Gap Law to the S_1_ Energies and Dynamics of Carotenoids. Chem. Phys. 1995, 194, 237–244. 10.1016/0301-0104(95)00017-I.

[ref101] Bensasson R.; Land E. J.; Maudinas B. Triplet States of Carotenoids from Photosynthetic Bacteria Studied by Nanosecond Ultraviolet and Electron Pulse Irradiation. Photochem. Photobiol. 1976, 23, 189–193. 10.1111/j.1751-1097.1976.tb07240.x. 817340

[ref102] Bensasson R.; Dawe E. A.; Long D. A.; Land E. J. Singlet → Triplet Intersystem Crossing Quantum Yields of Photosynthetic and Related Polyenes. J. Chem. Soc., Faraday Trans. 1 1977, 73, 1319–1325. 10.1039/f19777301319.

[ref103] Kollmar C. Electronic Structure of Diradical and Dicarbene Intermediates in Short-chain Polydiacetylene Oligomers. J. Chem. Phys. 1993, 98, 7210–7228. 10.1063/1.464713.

[ref104] Chattopadhyay S. K.; Das P. K.; Hug G. L. Photoprocesses in Diphenylpolyenes. 2. Excited-State Interactions with Stable Free Radicals. J. Am. Chem. Soc. 1983, 105, 6205–6210. 10.1021/ja00358a002.

[ref105] Fujii R.; Onaka K.; Kuki M.; Koyama Y.; Watanabe Y. The 2A_g_^–^ Energies of All-Trans-Neurosporene and Spheroidene as Determined by Fluorescence Spectroscopy. Chem. Phys. Lett. 1998, 288, 847–853. 10.1016/S0009-2614(98)00376-5.

