# Supplementary material for: Twisted Carotenoids Do Not Support Efficient Intramolecular Singlet Fission in the Orange Carotenoid Protein
Source: J Phys Chem Lett. 2023 Jun 26;14(26):6135–42. doi: 10.1021/acs.jpclett.3c01139 (PMC10331831; doi:10.1021/acs.jpclett.3c01139)
Supplement: Supplementary file 1 — jz3c01139_si_001.pdf [file jz3c01139_si_001.pdf]

Twisted Carotenoids Do Not Support Efficient Intramolecular Singlet Fission in the Orange Carotenoid Protein – Supplementary Information

George A. Sutherland,[†,‖] James P. Pidgeon,[‡,‖] Harrison Ka Hin Lee,[¶] Matthew S. Proctor,[†] Andrew Hitchcock,[†] Shuangqing Wang,[‡] Dimitri Chekulaev,[§] Wing Chung Tsoi,[¶] Matthew P. Johnson,[†] C. Neil Hunter,[†] and Jenny Clark[*,‡]

†*Plants, Photosynthesis and Soil, School of Biosciences, University of Sheffield, Sheffield S10 2TN, UK*

‡*Department of Physics and Astronomy, University of Sheffield, Sheffield S3 7RH, UK*

¶*SPECIFIC, Faculty of Science and Engineering, Swansea University, Swansea SA1 8EN, UK*

§*Department of Chemistry, University of Sheffield, Sheffield S3 7HF, UK*

‖*These authors contributed equally.*

E-mail: jenny.clark@sheffield.ac.uk

Phone: +44 (0)114 222 3526

Contents

S1 Materials and methods **S3**

S1.1 Sample preparation S3

S1.2 Steady-state absorbance spectroscopy S4

S1.3 Transient absorption spectroscopy S4

S1.4 Resonance Raman spectroscopy S5

S1.5 Figure preparation S6

S2 Additional steady-state absorbance data **S7**

S3 Additional resonance Raman data **S8**

S4 Additional transient absorption data **S10**

S4.1 Pump spectrum S10

S4.2 Global lifetime analysis S11

References **S15**

S1 Materials and methods

S1.1 Sample preparation

OCP containing ∼100% canthaxanthin (CAN) was produced from BL21(DE3) *Escherichia coli* (*E. coli*) using a dual-plasmid system comprised of pAC-CANTHipi [1] and pET28a containing the gene encoding OCP (slr1963) from *Synechocystis* sp. PCC 6803. Briefly, 500 mL cultures were grown at 37 °C (200 rev min^{-1} agitation) in 2 L baffled Erlenmeyer flasks using lysogeny broth (LB) medium containing the appropriate concentrations of antibiotics. When the absorbance of the medium at 600 nm had reached $A_{600} = 0.6$ (1 cm path length), protein production was induced by addition of 0.5 mM isopropyl β-D-1-thiogalatopyranoside and the cultures incubated for 16 hours at 18 °C.

Cells were harvested by centrifugation (4,400×g, 30 min, 4 °C) and resuspended in binding buffer (50 mM HEPES, pH 7.4, 500 mM NaCl, 5 mM imidazole). Cells were lysed by sonication and then centrifuged (53,000×g, 30 min, 4 °C). The supernatant was collected and filtered (0.22 µm filter pores) and applied to a Chelating Sepharose Fast Flow column (GE Healthcare) pre-equilibrated with $NiSO_4$. The column was washed with binding buffer, wash buffer (50 mM HEPES, pH 7.4, 500 mM NaCl, 50 mM imidazole) and elution buffer (50 mM HEPES, pH 7.4, 100 mM NaCl, 400 mM imidazole) with the elution pooled for further purification. The protein sample was buffer exchanged into buffer A (50 mM HEPES, pH 7.4) loaded onto a Fast Flow Q-Sepharose column (GE Healthcare) and a linear gradient of 0–1 M NaCl was applied. Fractions were analyzed by SDS-PAGE and appropriate samples taken forward for size exclusion chromatography on a Superdex 200 Increase column (GE Healthcare) in buffer B (50 mM HEPES, pH 7.4, 200 mM NaCl). Where necessary OCP samples were concentrated using centrifugal dialysis (VivaSpin, Sartorius).

OCPo samples were fixed in sucrose-trehalose glasses in strict darkness by mixing 100 µL of concentrated protein solution ($A_{max} \sim 2$, 1 cm path length) in aqueous buffer (50 mM HEPES, 200 mM NaCl, pH 7.4) with 100 µL of a trehalose-sucrose mixture (0.5 M trehalose,

0.5 M sucrose). 200 µL of the protein-trehalose mixture was drop-cast in the center of a quartz-coated glass substrate (S151, Ossila; 15×20×1.1 mm). The substrate was incubated under vacuum (−70 kPa) with an excess of calcium sulfate desiccant (Drierite) at room temperature for at least 48 hours. OCPr trehalose glasses were made in an identical manner, with samples illuminated for 30 min (1600 µmol photon m^{-2} s^{-1}) prior to the addition of the trehalose-sucrose solution and constant weaker illumination (500 µmol photon m^{-2} s^{-1}) for the duration of the desiccation.

OCPo and OCPr samples measured with the resonance Raman setup were additionally encapsulated with imaging spacers and a cover slip to protect the trehalose against atmospheric rehydration. For these samples, a stack of two imaging spacers (SecureSeal, Grace BioLabs; 9 mm diameter, 0.12 mm thickness) were attached to the quartz-coated glass substrate (S151, Ossila; 15×20×1.1 mm) and 40 µL of the protein-trehalose mixture drop-cast in the center of the imaging spacer. The substrate was then placed in vacuum as above; pressure was released under a continuous flow of ultra-pure nitrogen gas and a glass microscope cover slip (ThermoScientific; 22×22 mm, No.1 thickness) was attached to the upper imaging spacer.

S1.2 Steady-state absorbance spectroscopy

Steady-state absorbance of samples were measured in a commercial Cary double-beam spectrometer (Cary 60 UV-Vis Spectrophotometer, Agilent Technologies). Both zero and baseline corrections were applied with a blank sample; in the case of trehalose-encapsulated samples, a trehalose blank is used.

S1.3 Transient absorption spectroscopy

Picosecond transient absorption spectroscopy was undertaken with a commercial spectrometer (Helios, Ultrafast Systems) outfitted with a Ti:Sapphire seed laser (MaiTai, Spectra Physics) providing 800 nm pulses (84 MHz, 25 fs nominal FWHM) and a Ti:Sapphire chirped-

pulse amplifier (Spitfire Ace PA-40) amplifying 800 nm pulses (10 kHz, 12 W average power, 40 fs nominal FWHM). Tunable pump pulses for excitation were generated by seeding a part of the 800 nm beam in an optical parametric amplifier (TOPAS Prime, Light Conversion). An optical chopper was used to modulate the pump frequency to 5 kHz. An intensity spectrum for the pump used in visible-probe measurements is shown in Figure S4. Pump beam spot sizes were measured at the sample position with a CCD beam profiler (BC106N-VIS/M, Thorlabs), and used in subsequent calculations to tune a 200 µJ cm^{-2} pump fluence. Supercontinuum probes were generated with a part of the 800 nm pulse focused on either a sapphire crystal for visible probes (450–800 nm) or a YAG crystal for NIR probes (800–1600 nm). Pump-probe delay was controlled with a motorized delay stage with a random stepping order. The signal was dispersed with a grating and detected with CMOS or InGaAs sensors for visible or NIR probes, respectively. The pump and probe polarizations were set to the magic angle.

Surface Xplorer 4.3.0 (Ultrafast Systems) was used in processing the transient absorption datasets. Noisy edges of the spectra were trimmed, and the program's bad spectra replacement procedure was applied. A background correction ('subtract scattered light') was then applied using the spectra before any apparent response from the sample. For visible-probe data, chirp correction was applied, choosing points at the first apparent signal for a given kinetic. For NIR-probe data, no chirp was discernible, hence no chirp correction was applied. Time zero is adjusted to the time of maximum initial signal. Further processing and some analysis was performed with original Python code.

S1.4 Resonance Raman spectroscopy

Resonance Raman measurements were performed with a Renishaw inVia Raman system (Renishaw plc., Wotton-Under-Edge, UK) in a backscattering configuration. A 532 nm laser (150–750 µW power) and a 50× objective were used (NA 0.50, spot size ∼1 µm). Acquisition times used were in the range 5–30 s.

S1.5 Figure preparation

OriginPro 9.6.0.172 (OriginLab) and home-built Python code was used to prepare the plots.

S2 Additional steady-state absorbance data

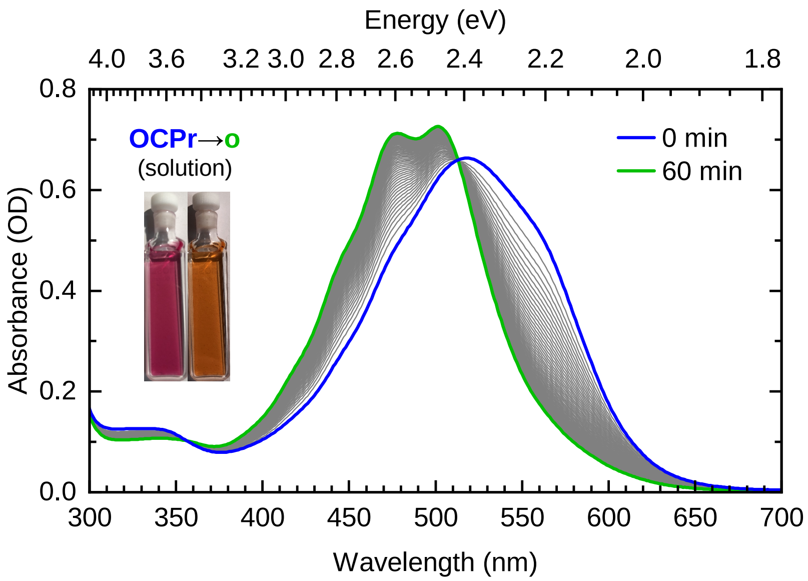

Figure S1: **Backconversion of OCPr to OCPo in solution.** Steady-state absorbance spectra of OCPr in solution taken in 1 min intervals at 22 °C in darkness. The optical path length was 1 mm.

S3 Additional resonance Raman data

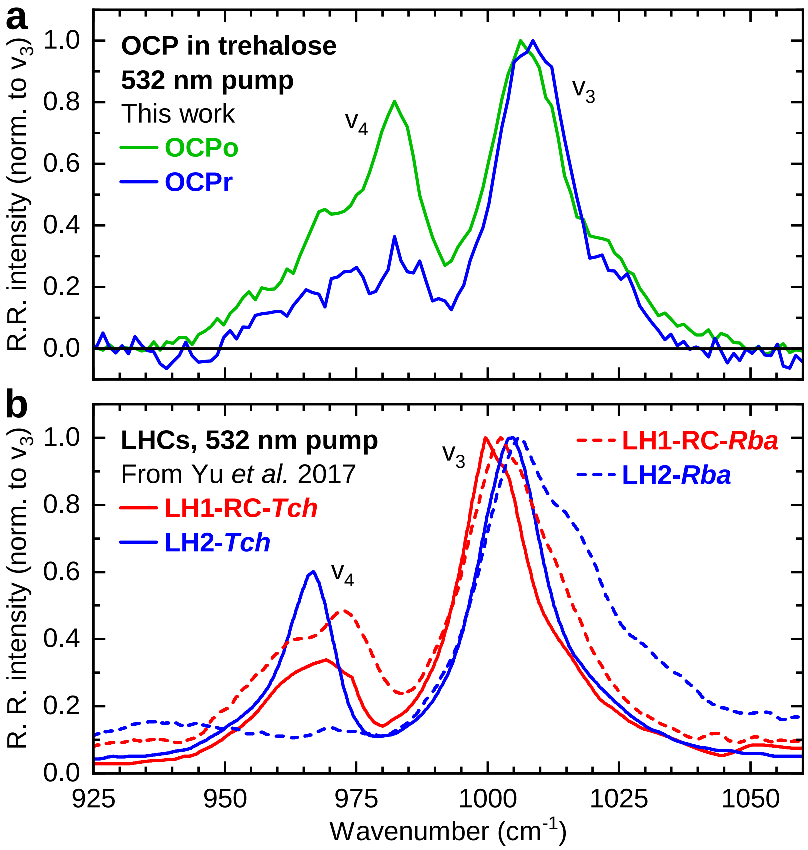

Figure S2: **Resonance Raman spectra of OCP in trehalose glass films and of purple bacterial LHCs.** A 532 nm pump was used in all experiments here, with normalization to the peak ν_3 intensity. (a) The spectra of OCPo (green) and OCPr (blue) are reproduced from Figure 2c, zoomed to the ν_3 and ν_4 peaks. Data is averaged from two successive scans. (b) The spectra of LHCs from *Thermochromatium tepidum* (LH1-RC-*Tch*, LH2-*Tch*) and *Rhodobacter sphaeroides* 2.4.1. (LH1-RC-*Rba*, LH2-*Rba*) are adapted with permission from Yu *et al.*[2] (copyright 2017 American Chemical Society). Note that LH2-*Rba* has no discernible ν_4 peak.

A comparison of the resonance Raman spectra of OCPo/OCPr in trehalose films of this work against the spectra of purple bacterial light-harvesting complexes (LHCs) from Yu *et al.*[2] is shown in Figure S2. The carotenoid ν_3 peak arises from CH_3 rocking in the plane of carotenoid conjugation, while the ν_4 peak arises from C−H wagging modes out of the plane of conjugation.[2–4] Therefore, the ν_4:ν_3 ratio is indicative of the degree of carotenoid backbone twisting.[2] For OCP binding canthaxanthin (CAN) in trehalose in panel (a), we see

that OCPo has a greater ν_4:ν_3 ratio, indicating that the CAN in OCPo is twisted while the CAN in OCPr is relatively planar. We do not see any evidence of efficient intramolecular singlet fission (iSF) in either OCPo or OCPr; see main text for details. For purple bacterial LHCs in panel (b), the LH2 from *Rhodobacter sphaeroides* 2.4.1. (LH2-*Rba*) binding >90% spheroidene[2] has no discernible ν_4 peak, so its ν_4:ν_3 ratio is near-zero and the carotenoids bound within LH2-*Rba* are said to be planar.[2] As LH2-*Rba* was the only LHC studied by Yu *et al.* showing no singlet fission (SF) contribution to its triplet population, the authors hypothesized that the carotenoid twist is the determinant for SF through an intramolecular scheme (*i.e.* through iSF).[2] This is counter to the lack of iSF in OCPo; see main text for details.

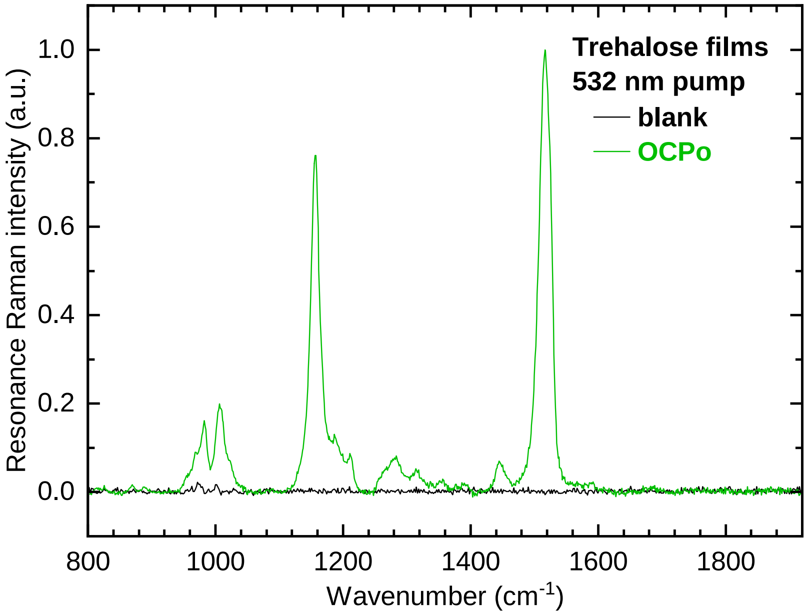

Figure S3: **Resonance Raman spectra of blank trehalose glass and OCPo in trehalose.** The blank trehalose spectrum (black) is almost entirely noise, confirming that the trehalose-sucrose glass is not contributing significant Raman signal in any of the samples incorporating trehalose glass. OCPo in trehalose (green) is shown as a comparison, normalized to the peak ν_1 intensity. The blank spectrum has been scaled to give a similar noise magnitude at the high-wavenumber end. Both spectra are single scans (not averaged). Measurements were undertaken at room temperature. A 532 nm pump was used.

S4 Additional transient absorption data

S4.1 Pump spectrum

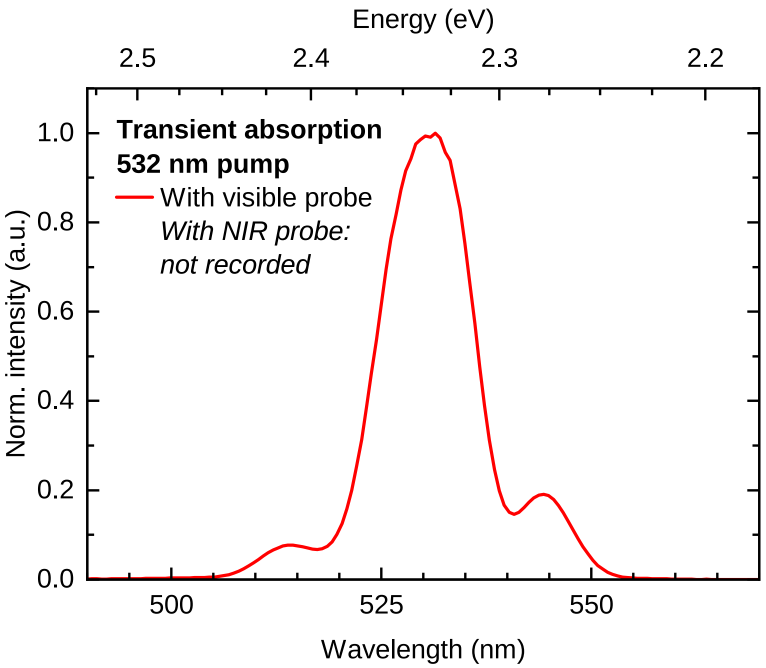

Figure S4: **Intensity spectrum of the pump used in the transient absorption experiments while using the visible probe.** This 532 nm-centered (set in WinTopas4) excitation profile was used in taking the visible-probe data shown in main text Figure 3 and Figure 4. The 532 nm-centered profile used in taking the NIR-probe data was not taken, and may have had a slightly different spectrum owing to day-to-day variation in the tunable pump generation. The spectrum has been normalized to the maximum intensity.

S4.2 Global lifetime analysis

Global lifetime analysis on the visible-probe transient absorption data of OCPo and OCPr was performed. This was done using the Glotaran 1.5.1 software package (http://glotaran.org),[5] a GUI for the R package TIMP.[6] Data used had already been processed with the steps outlined in Section S1.3; in particular, a chirp correction had already been applied, so that a term to account for chirp did not need to be included in the fitting. Noisy regions in the data due to pump scatter were excluded for all times to ensure a good fit of the rest of the data. Noisy red and blue ends in the data associated with tails of the probe were also excluded, so that the fitted wavelengths were 430 nm to 780 nm. The fitting was weighted favourably at later delay times for good fits of any long-lived features; Table S1 shows the weighting applied. Due to the strong coherent artifact feature in the first 0.5 ps, only data beyond that time was fitted. Thus, in the model, terms to account for the coherent artifact and S_2 states were not included. This left a relatively simple fitted model of a number of wavelength-dependent decay-associated difference spectra (DADS) decaying exponentially in parallel.

Table S1: **Weightings applied to time-ranges of the visible ps transient absorption data for the global lifetime analysis.** Note that only data >0.5 ps was fitted, and that the maximum time delay in these experiments was ~40 ps.

**Time range (ps)**	**Weighting**
0.5 – 10	1
10 – 20	2
20 – 30	3
30 – 35	4
>35	5

Global lifetime analysis of the OCP data does not indicate any significant population of longer-lived features. 2-component global lifetime analysis of the CAN-binding OCPo and OCPr data are shown in Figure S5 and S6, respectively. Fitting a 2-component parallel decay model in an artifact-free region of the visible-probe data beyond the initial coherent

artifact and S_2-associated response gives two decay-associated difference spectra (DADS) for both the OCPo data and OCPr data, with the longer time-constant DADS relatively weaker and blueshifted in both cases. Both components are likely associated with mixed S_1 decay, internal vibrational redistribution, and vibrational cooling.[7] A single component is not sufficient to adequately fit the region of the data, and a fitting a third component gives results with spurious DADS profiles.

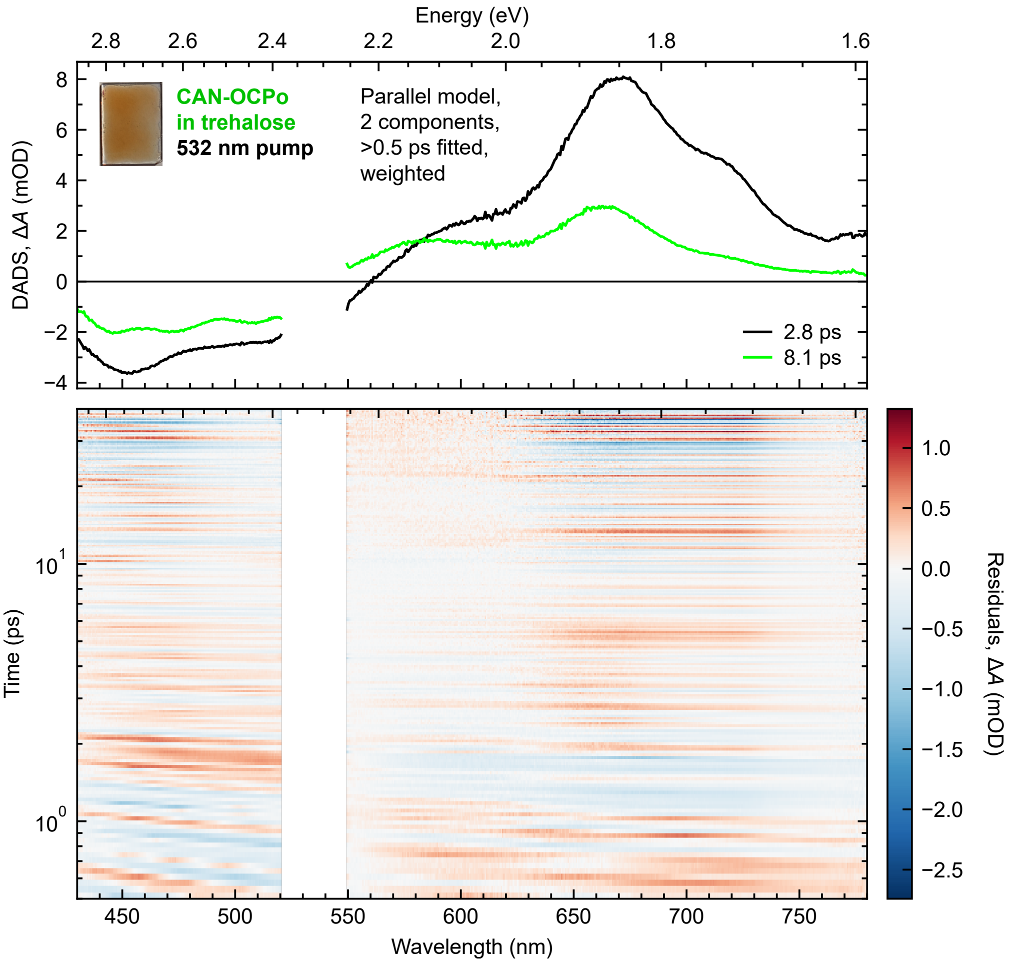

Figure S5: **Results of global lifetime analysis with a 2-component parallel model on transient absorption data of CAN-binding OCPo in trehalose with peak pump wavelength 532 nm and a visible probe: DADS (top) and residuals (bottom).** Only the wavelength range 430–780 nm and times >0.5 ps were fitted, and noisy data from 520.5–549.5 nm due to significant pump scatter was excluded from the fit. DADS lifetimes are specified in the legend. Residuals = Data − Fit. See text for further details.

We note that sample degradation (caused the ∼200 μJ cm^{-2} pump fluence) likely affects

the fitted time constants and DADS profiles, and that the maximum time delay used was about 40 ps.

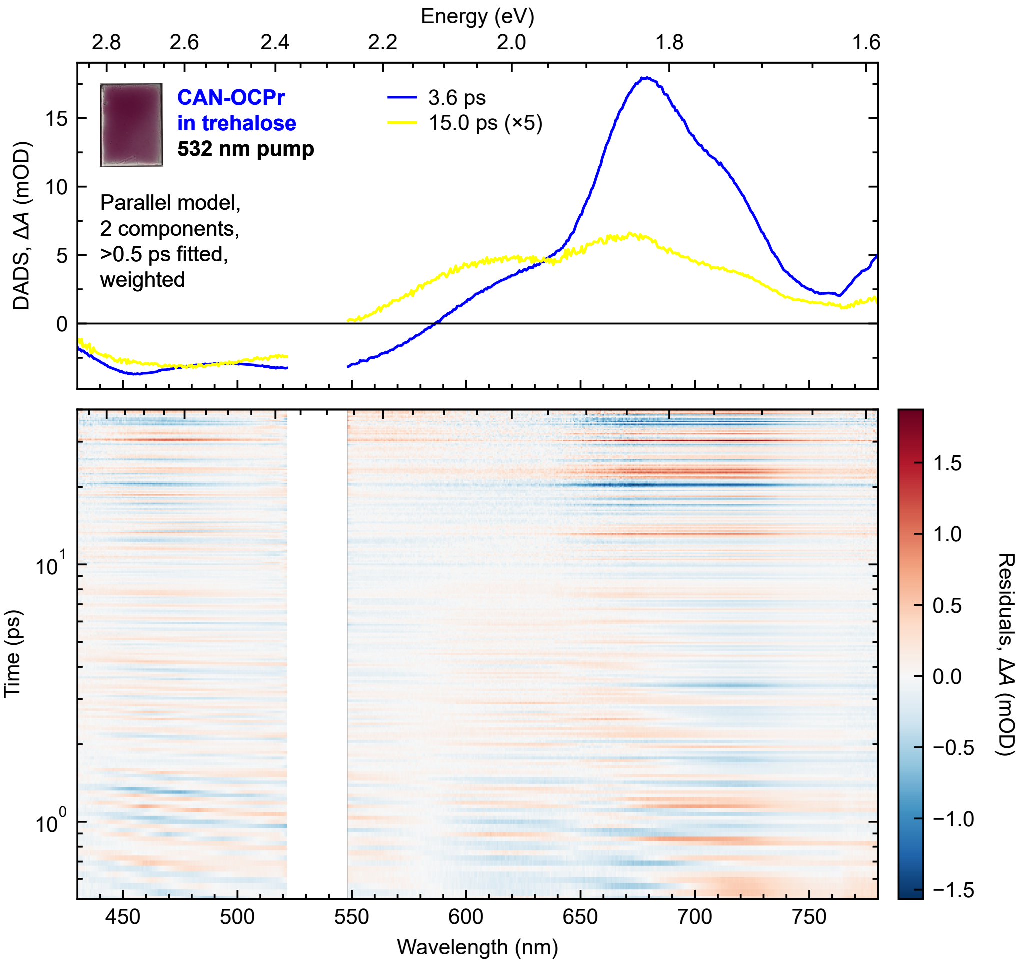

Figure S6: **Results of global lifetime analysis with a 2-component parallel model on transient absorption data of CAN-binding OCPr in trehalose with peak pump wavelength 532 nm and a visible probe: DADS (top) and residuals (bottom).** Only the wavelength range 430–780 nm and times >0.5 ps were fitted, and noisy data from 522–548 nm due to significant pump scatter was excluded from the fit. DADS lifetimes are specified in the legend; multiplications refer to scalings applied to the DADS. Residuals = Data − Fit. See text for further details.

References

(1) Cunningham, F. X.; Gantt, E. A Portfolio of Plasmids for Identification and Analysis of Carotenoid Pathway Enzymes: *Adonis aestivalis* as a Case Study. *Photosynth. Res.* **2007**, *92*, 245–259.

(2) Yu, J.; Fu, L.-M.; Yu, L.-J.; Shi, Y.; Wang, P.; Wang-Otomo, Z.-Y.; Zhang, J.-P. Carotenoid Singlet Fission Reactions in Bacterial Light Harvesting Complexes As Revealed by Triplet Excitation Profiles. *J. Am. Chem. Soc.* **2017**, *139*, 15984–15993.

(3) Saito, S.; Tasumi, M. Normal-Coordinate Analysis of Retinal Isomers and Assignments of Raman and Infrared Bands. *J. Raman Spectrosc.* **1983**, *14*, 236–245.

(4) Kish, E.; Pinto, M. M. M.; Kirilovsky, D.; Spezia, R.; Robert, B. Echinenone Vibrational Properties: From Solvents to the Orange Carotenoid Protein. *Biochim. Biophys. Acta, Bioenerg.* **2015**, *1847*, 1044–1054.

(5) Snellenburg, J.; Laptenok, S.; Seger, R.; Mullen, K. Glotaran: A Java-Based Graphical User Interface for the R Package TIMP. *J. Stat. Softw.* **2012**, *49*.

(6) Mullen, K. M.; van Stokkum, I. H. M. TIMP: An R Package for Modeling Multi-Way Spectroscopic Measurements. *J. Stat. Softw.* **2007**, *18*, 1–46.

(7) Balevičius, V.; Wei, T.; Di Tommaso, D.; Abramavicius, D.; Hauer, J.; Polívka, T.; Duffy, C. D. P. The Full Dynamics of Energy Relaxation in Large Organic Molecules: From Photo-Excitation to Solvent Heating. *Chem. Sci.* **2019**, *10*, 4792–4804.
